# Biomedical informatics with optimization and machine learning

**DOI:** 10.1186/s13637-017-0058-0

**Published:** 2017-02-08

**Authors:** Shuai Huang, Jiayu Zhou, Zhangyang Wang, Qing Ling, Yang Shen

**Affiliations:** 1 Department of Industrial and Systems Engineering, University of Washington, Seattle, WA 98195 USA; 2 Department of Computer Science and Engineering, Michigan State University, East Lansing, MI 48824 USA; 3 Department of Computer Science and Engineering, Texas A&M University, College Station, TX 77843 USA; 4 Department of Automation, University of Science and Technology of China, Hefei, Anhui 230026 China; 5 Department of Electrical and Computer Engineering and TEES-AgriLife Center for Bioinformatics and Genomic Systems Engineering, Texas A&M University, College Station, TX 77843 USA

Fast-growing biomedical and healthcare data have encompassed multiple scales ranging from molecules, individuals, to populations and have connected various entities in healthcare systems (providers, pharma, payers) with increasing bandwidth, depth, and resolution. Those data are becoming an enabling resource for accelerating basic science discoveries and facilitating evidence-based clinical solutions. Although the methods for extracting patterns from data have been around for centuries, it is still extremely difficult to transform massive data into valuable knowledge by these traditional means of analysis. This motivates the development of modern analytics methods, which are designed to discover meaningful representations or structures of data using optimization and machine-learning methods. In a broad sense, there are two types of applications in biomedical informatics where optimization and machine-learning methods are commonly used. One focuses on the knowledge discovery by analyzing historical data to provide insights on what happened and why it happened. Methods such as data statistical modeling, trend reporting, and visualization as association and correlation analysis have been commonly used in this sort of applications. Another sort of applications, on the other hand, focus on prediction and decision-making applications that use a known dataset (aka the training dataset), and which includes input data features and response values, to build a predictive model and scale it to make predictions using unseen data (aka the test dataset).

It has been a consensus that the sheer volume and complexity of the data we could easily acquire nowadays in biomedical informatics present major barriers toward their translation into effective clinical actions. There is thus a compelling demand for novel algorithms, including machine learning, data mining, and optimization that specifically tackle the unique challenges associated with the biomedical and healthcare data and allow decision-makers and stakeholders to better interpret and exploit the data. Recent years have witnessed major breakthroughs in machine learning when it is equipped with powerful optimization technologies. On a general note, biomedical data often feature large volumes, high dimensions, imbalanced classes, heterogeneous sources, noisy data, incompleteness, and rich contexts. Such demanding features are also driving the development of numerical optimization algorithms in tandem with machine learning algorithms. For example, it has been a challenge to deal with roadblocks in the biomedical informatics area given the ubiquitous existence of data challenges such as imbalanced datasets, weakly structured or unstructured data, noisy and ambiguous labeling. Also, the optimization algorithms should scale up to the complexity of biomedical data that is usually large-scale, high-dimensional, heterogeneous, and noisy. It is also of much interest to study and revisit traditional machine-learning topics such as clustering, classification, regression, and dimension reduction and turn them into powerful customized approaches for the newly emerging biomedical informatics problems such as electronic medical records analysis and heterogeneous data fusion.

Besides the methodological issues, there are much to be learned through the application of these methods in real-world applications, regarding how the context of the applications informs the design, implementation, interpretation, and validation of these methods. Challenging applications are present in many areas of biomedical informatics, such as Computational Biology, which includes the advanced interpretation of critical biological findings, using databases and cutting-edge computational infrastructure; Clinical Informatics, which includes the scenarios of using computation and data for health care, spanning medicine, dentistry, nursing, pharmacy, and allied health; Public Health Informatics, which includes the studies of patients and populations to improve the public health system and to elucidate epidemiology; mHealth Applications, which include the use of mobile apps and wearable sensors for health management and wellness promotion; and Cyber-Informatics Applications, which include the use of social media data mining and natural language processing for clinical insight discovery and medical decision making. For building a predictive model, predictive analytics deals with the problems associated with the identification and removal of superfluous information present in a dataset, a task referred to as feature selection. Feature selection is needed for managing the dimensionality of the dataset, which grows with the number of features. More specifically, it reduces the dimensionality of data by selecting only a subset of data features to create a decision model. In addition to dimensionality reduction, feature selection is also closely related to overfitting. Here, overfitting refers to the common risk of machine-learning models that may fit the noise rather than the signal of interest. Having a minimal number of features often leads to simpler models, better generalization, and easier interpretation. The concept of parsimony (Occam’s razor) is often invoked to bias the search: never do more with more than what can be done with less. Feature selection criteria usually involve the minimization of a specific predictive error measure for model fitting to different data subsets. In recent years, sparse learning (aka regularization) has gained popularity as an integrated learning method for simultaneously selecting features and building classification models. All these issues represent general traits and guiding principles for the papers included into this journal special issue.

The goal of this special issue is to present state-of-the-art and emerging machine learning and optimization methods that deal with the above-mentioned real-world challenges in biomedical informatics. This special issue consists of eight papers that treat important machine learning and optimization topics in biomedical informatics topics such as prediction problems in protein-protein interaction, electronic medical record data mining, health question answering, text mining from public medical knowledge repositories, prognosis of carotid atherosclerosis patients, detection of disease symptoms from face information, detection of autism spectrum disorder from medical data, and heterogeneous biomarker analysis for understanding progression of Alzheimer’s disease, using a wide range of methods such as principal component analysis, naïve Bayes classifier, random forest, sparse learning, information theory-based machine learning, text mining, Bayesian network, information retrieval, and computer vision algorithms. A short description of the contributions brought by the papers of this special issue is next presented.

In the paper *Stochastic Convex Sparse Principal Component Analysis*, Inci Baytas, Kaixiang Lin, Fei Wang, Anil Jain, and Jiayu Zhou deal with the important problem of interpretability of Principal component analysis (PCA) in medical applications. As the conventional PCA methods generate principal components which are linear combinations of all the original features, it results in commonly known challenges in interpretation: if one attempts to identify significant variables that constitute the principal components or correlate the statistical significance with physical knowledge. Thus, these authors proposed herein paper a new method to conduct sparse PCA that scales up well for large-scale applications by exploiting a stochastic gradient framework which can achieve a geometric convergence rate. The method is showcased on a large-scale electronic medical record dataset, which proves its utility in real-world biomedical informatics applications.

In the paper *Towards Organizing Health Knowledge on Community-based Health Services*, Mohammad Akbari, Xia Hu, Liqiang Nie, and Tat-Seng Chua propose a top-down organization scheme, which can automatically assign the unstructured health-related records into a hierarchy with prior domain knowledge. With the accumulation of unstructured health question answering (QA) records, the ability to organize them has been found to be effective for data access. Existing approaches are often not applicable to the health domain due to its domain nature as characterized by the complex relations among entities, large vocabulary gap, and heterogeneity of users. The authors of this paper design a hierarchy-based health information retrieval system. Experiments carried out on a real-world dataset demonstrate the effectiveness of the proposed scheme in organizing health QA records into a topic hierarchy and retrieving health QA records from the topic hierarchy.

In the paper *Complex Temporal Topic Evolution Modelling using the Kullback-Leibler Divergence and the Bhattacharyya Distance*, Victor Andrei and Ognjen Arandjelović present advanced machine-learning techniques to automatically understand previous medical research literature, extract maximum information from the collected datasets, and identify promising research directions. The proposed framework is based on (i) the discretization of time into epochs, (ii) epoch-wise topic discovery using a hierarchical Dirichlet process-based model, and (iii) a temporal similarity graph which allows for the modeling of complex topic changes. The proposed machine learning techniques are also evaluated on a public medical literature corpus. This is the first work that discusses and distinguishes between two groups of particularly challenging topic evolution phenomena: topic splitting and speciation, and topic convergence and merging, in addition to the more widely recognized emergence, disappearance, and gradual evolution.

In the paper *Detecting Visually Observable Disease Symptoms from Faces*, Kuan Wang and Jiebo Luo present a generalized solution to detect visually observable symptoms present on faces using semi-supervised anomaly detection combined with machine vision algorithms. Recent years have witnessed an increasing interest in the application of machine learning to clinical informatics and healthcare systems. A significant amount of research has been done on healthcare systems based on supervised learning. The proposed approach relies on the disease-related statistical facts to detect abnormalities and classify them into multiple categories to narrow down the possible symptoms. Experiments verify the major advantages of the proposed solution in flagging unusual and visually observable symptoms.

In the paper *Enhancing Interacting Residue Prediction with Integrated Contact Matrix Prediction in Protein–Protein Interaction*, Tianchuan Du, Li Liao, and Cathy Wu delve into the molecular level and develop a combined framework to solve two related tasks about proteins: interaction site prediction and contact matrix prediction. They combined predictions for interaction sites from an interaction profile hidden Markov model (ipHMM) and predictions for contact matrices from support vector machines based on the derived ipHMM and other features. Furthermore, these authors integrated these predictions as features into a logistic regression model to improve the interaction site prediction. The hierarchical use of the predictor-generated features and the integration of features provide an integrated and improved way to address the problem.

In the paper *Machine Learning to Predict Rapid Progression of Carotid Atherosclerosis in Patients with Impaired Glucose Tolerance*, Xia Hu, Peter Reaven, Aramesh Saremi, Ninghao Liu, Mohammed Abbasi, Huan Liu, and Raymond Q. Migrino study the important problem of predicting the rapid progression of carotid intima-media thickness in impaired glucose tolerance participants. These authors study the important factors impacting the prediction by employing a probabilistic Bayes method and several other competing methods. The experimental results carried out on the real-world ACT NOW dataset corroborate the effectiveness of the proposed computational framework.

In the paper *Autism Spectrum Disorder Detection from Semi-Structured and Unstructured Medical Data*, Jianbo Yuan, Chester Holtz, Tristram H. Smith, and Jiebo Luo propose a method for detecting autism spectrum disorder (ASD) from medical records (usually unstructured/semi-structured data sets) by resorting to classification machine-learning techniques. Since the diagnosis of ASD could be labor-intensive, time consuming, and might require extensive expertise, the authors proposed a data-driven method (based on the existing medical records) to assist the diagnosis of ASD. The experimental results are solid and could be helpful in clinical decisions.

In the paper *Heterogeneous Multimodal Biomarkers Analysis for Alzheimer’s Disease via Probabilistic Bayesian Network*, Yan Jin, Yi Su, Xiao-Hua Zhou, and Shuai Huang applied a mixed-type Bayesian network learning technique to multiple candidate biomarkers collected in ADNI for Alzheimer’s disease to understand the association of these candidate biomarkers with the disease progression and the underlying disease mechanisms. Specific technical challenges that were addressed in this paper include the handling of mixed types of biomarker data, categorical and numerical, and providing a systematic understanding of the relationships between these data through Bayesian network modeling. The proposed Bayesian network model yields findings that are consistent with the existing Alzheimer’s disease literature, and deliver great prediction accuracy of the clinical outcomes.

